# The Thermomechanical Properties of Thermally Evaporated Bismuth Triiodide Thin Films

**DOI:** 10.1038/s41598-019-48194-1

**Published:** 2019-08-13

**Authors:** Natália F. Coutinho, Silvia Cucatti, Rafael B. Merlo, José Maria C. Silva Filho, Nelson F. Borrero Villegas, Fernando Alvarez, Ana F. Nogueira, Francisco C. Marques

**Affiliations:** 1 “Gleb Wataghin” Institute of Physics - University of Campinas (UNICAMP), Campinas, SP 13083-859 Brazil; 2 Institute of Chemistry - University of Campinas (UNICAMP), Campinas, SP 13083-970 Brazil

**Keywords:** Surfaces, interfaces and thin films, Semiconductors, Solar cells

## Abstract

Bismuth triiodide (BiI_3_) has been studied in recent years with the aim of developing lead-free semiconductors for photovoltaics. It has also appeared in X-ray detectors due to the high density of the Bismuth element. This material is attractive as an active layer in solar cells, or may be feasible for conversion into perovskite-like material (MA_3_Bi_2_I_9_), being also suitable for photovoltaic applications. In this study, we report on the thermomechanical properties (stress, hardness, coefficient of thermal expansion, and biaxial and reduced Young’s moduli) of BiI_3_ thin films deposited by thermal evaporation. The stress was determined as a function of temperature, adopting the thermally induced bending technique, which allowed us to extract the coefficient of thermal expansion (31 × 10^−6^ °C^−1^) and Young’s biaxial modulus (19.6 GPa) for the films. Nanohardness (~0.76 GPa) and a reduced Young’s modulus of 27.1 GPa were determined through nanoindentation measurements.

## Introduction

The current demand for cost-efficient and renewable materials for energy applications has recently acted as a great driving force towards the development of low cost photovoltaic cells. The majority of commercial solar cells currently use crystalline silicon as the base semiconductor; in all probability, the most promising material for future solar generation will be one based on perovskite, this possessing efficiencies as high as 23.7%^1^. In spite of the surprising efficiency obtained, perovskite-based solar cell production faces problems related to stability, quite apart from the use of lead, a toxic element. With a view to overcoming this issue, some lead-free materials suitable for photovoltaic applications have gained the attention of researchers in recent years. In their studies, bismuth triiodide (BiI_3_)^2–5^ has revealed itself to be a promising candidate for photovoltaic applications, being a semiconductor with an adequate band gap for these purposes: 1.67 eV^6^. This material is versatile, and useful as the active absorbent layer in solar cells; it may also be suitable for conversion into a perovskite-like material such as MA_3_Bi_2_I_9_, which is additionally suitable for photovoltaic applications^7–9^. The efficiencies of devices using MA_3_Bi_2_I_9_ as absorber material are still very low (1.64%)^9^ and therefore optimization studies need to be performed to increase these values. In spite of this, the use of BiI_3_ in combination with PbI_2_ as precursors for a more stable perovskite CsPb_x_Bi_y_I_3_ provided an efficiency of 11.47% in flexible substrates^10^.

In addition to the issue of the chemical stability of the semiconductors used in solar cell devices, other solar cell problems are associated with interface issues between the various materials used in the device structure. A typical perovskite solar cell is composed of 5–6 layers of various materials with differing thermomechanical properties. The difference in the inter-layer’s thermal expansion causes stress, and thus cracking, to appear within its structure.

We recently reported some preliminary results on stress and nanoindentation measurements of thermally evaporated BiI_3_^11^. In this work, we employed the thermally-induced bending (TIB) technique^12^ and nanoindentation measurements^13^ to determine five thermomechanical properties: stress (σ), the coefficient of thermal expansion (CTE or α), nanohardness (H) and two elastic moduli related to Young’s modulus (E) and Poisson’s ratio (ν), namely, the biaxial Young’s modulus [E/(1 − ν)] and reduced Young’s modulus [E/(1 − ν^2^)]. There is little work addressing these subjects in photovoltaic-applied materials. Some of these properties have been reported for amorphous semiconductors^14–16^ and perovskite films deposited by solution processes^17,18^.

## Results and Discussion

### Optical and structural properties

**Figure 1:**
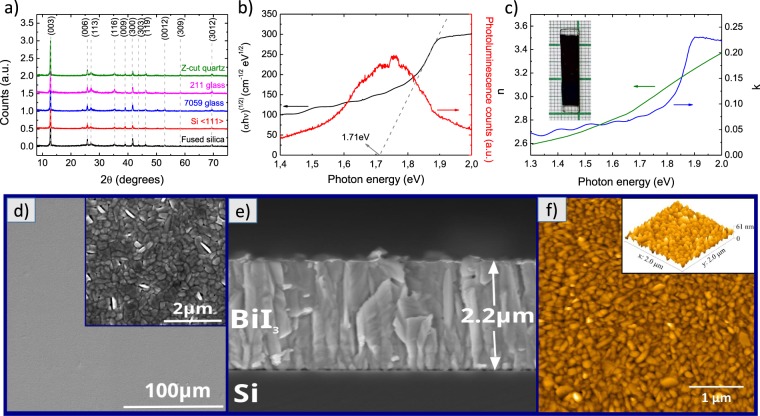
(**a**) X-ray diffraction pattern of bismuth triiodide thin films deposited on Z-cut quartz, 211 Precision glass, 7059 Corning glass, 〈111〉 silicon and fused silica (normalized at (003) peak). (**b**) Tauc’s plot (black) and photoluminescence spectra (red) of BiI_3_ thin films (the extrapolation of the curve to (αhν)^1/2^ = 0 and the center of the photoluminescence emission gives an estimated band gap of 1.71 eV and 1.74 eV, respectively). (**c**) Refractive index and extinction coefficient as a function of photon energy (the inset is a photo of a sample −25 mm × 5 mm in size). (**d**,**e**) Scanning electron microscope images of top view and cross section of BiI_3_ films, respectively and (**f**) AFM image.

Figure 1 shows the structural, morphological and optical properties of BiI_3_ films. The X-ray diffraction pattern (Fig. 1a) indicates that the films deposited on all substrates (Z-cut quartz, 〈111〉 silicon, 211 Precision glass, 7059 Corning glass and fused silica) have an R-3 rhombohedral crystal structure (JCPDS PDF # 48-1795)^19^ with a preferential orientation in the (003) direction. Figure 1b shows Tauc’s plot: (αhν)^1/r^ versus hν, where α and hν are, respectively, the optical absorption coefficient and incident photon energy. Considering *r* = 2 for indirect allowed transitions^20^, the extrapolation of the Tauc’s plot linear region for (αhν)^1/2^ = 0 shows an estimated band gap of 1.71 eV, while the center of the photoluminescence emission at room temperature results in a value of 1.74 eV. Our results agree with those of R. E. Brandt *et al*.^2^, who through the use of DFT incorporating spin-orbit coupling encountered an indirect band gap of 1.73 eV; and with those of Nikolas J. Podraza *et al*.^6^, who determined that a single BiI_3_ crystal has an indirect band gap of 1.67 eV. The latter reached this value after several measurements aimed at solving discrepancies found in the literature, which varied from a direct band gap of 2.2 eV^21^ to an indirect one of 1.55 eV^6^. UV-Vis measurements performed in monocrystalline and solution-processed BiI_3_ films supplied respective values of 1.68 eV^22^ and 1.8 eV^2^. The index of refraction and the extinction coefficient as a function of photon energy appear in Fig. 1c.

SEM images of thermally evaporated BiI_3_ films in Fig. 1(d,e) show a film 2.2 μm thick, homogeneously distributed along the substrate, and grain sizes of approximately 270 nm. Through AFM we were able to obtain a root mean square (RMS) roughness of 6 nm (Fig. 1f). Thicker and homogeneously distributed films are important parameters for both the stress and coefficient of thermal expansion measurements – see the description of said technique in Methods section. These characteristics are easy to obtain by using thermal evaporation, although the same cannot be said for spin coating^23^.

### Stress, coefficient of thermal expansion and biaxial Young’s Modulus

**Figure 2:**
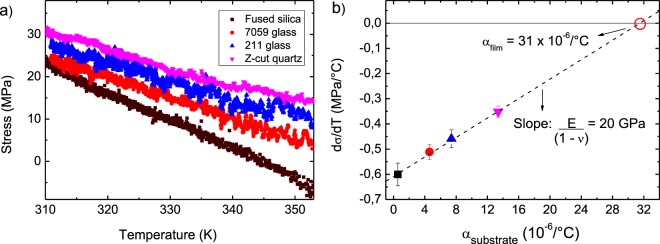
(**a**) Typical stress versus temperature curves of BiI_3_ deposited on fused silica, 7059 Corning glass, 211 Precision glass and Z-cut quartz substrates; (**b**) derivatives of stress, extracted from (**a**), with respect to temperature (dσ/dT) of BiI_3_ versus CTE (or α) of the substrates. The data are average of measurements performed in five films deposited in each substrate at different runs. The CTE and biaxial Young’s modulus of BiI_3_ were extracted from the indicated fitting.

We measured the film stress (σ) as a function of temperature in several substrates – see Fig. 2a. The thermally evaporated BiI_3_ films are tensile (positive stress) with an average stress of 28 ± 3 MPa at 300 K. This low value might be associated with thermal stress due to a non-intentional increase in the substrate temperature during film growth, which is caused by irradiation from the tungsten boat. Low stress values of films used in electronic devices, such as solar cells, are desirable because it prevents degradation due to stresses in the structure of the devices. Typical amorphous semiconductors used in these devices, such as amorphous silicon and germanium, usually demonstrate compressive stress of at least one order of magnitude higher^14,15,24^. On the other hand, perovskite films like Cs_x_FA_1−x_Pb(Br_y_I_1−y_)_3_ has stress within the same range as the one here obtained for BiI_3_^17^.

Using only two substrates, it is possible to solve the linear system of two Eq. (2) (one for each substrate), calculate the biaxial Young’s modulus E_f_/(1−ν_f_) and CTE of BiI_3_. A graphical procedure to calculate these parameters, when more than two substrates are used, is displayed in Fig. 2b, which consists of plotting dσ/dT obtained from the derivative of curves shown in Fig. 2a as a function of substrate CTE^12^. The data slope is the biaxial Young’s modulus, with the intersection with the CTE axis (dσ/dT = 0) being film CTE. All data in Fig. 2b is an average of five samples with films approximately 2.2 μm thick for each substrate (Fig. 1e). Through these results we obtained a biaxial Young’s modulus of 19.6 ± 0.8 GPa and a CTE of 31 ± 1 (10^−6^ °C^−1^) for BiI_3_ thin films.

The CTE of glass substrates are typically in the 3–6 × 10^−6^ °C^−1^ range, being much smaller than that determined here for BiI_3_. Thus, solar cells or other electronic devices based on BiI_3_ deposited on glass may suffer degradation with time due to the marked mismatch between their thermal expansions. Conversely, a high value of BiI_3_ CTE makes it suitable for deposition onto polymeric substrates, since they possess high CTE^25^. Intrinsic stress or stress due to lattice mismatches may be reduced by the use of a buffer layer^26,27^. It is likely that with this procedure one is able to reduce the effect of stress due to a difference in thermal expansion. The inclusion, however, of an additional layer in electronic devices requires careful control of its effect in the device’s performance. Typical values for the coefficient of thermal expansion of conventional amorphous semiconductors (amorphous silicon and germanium) are in the 1–10 × 10^−6^ °C^−1^ range^12,15^, which is much smaller than the one obtained here for BiI_3_. As far as we are aware, no data for perovskite thin films have been reported yet.

### Hardness and reduced Young’s Modulus

**Figure 3:**
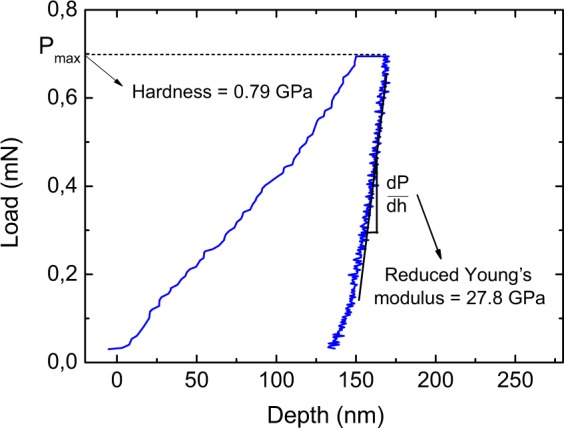
Typical nanoindentation curve of a 2.2 μm-thick BiI_3_ film deposited on 211 Precision glass.

Figure 3 shows a typical load-displacement curve obtained from nanoindentation measurements performed on a BiI_3_ film. For this single measurement, one obtains a reduced Young’s modulus of 27.8 GPa and a hardness of 0.79 GPa.

**Figure 4:**
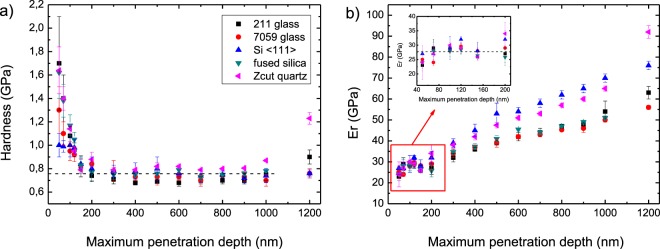
Maximum penetration depth dependence of hardness (**a**) and reduced Young’s modulus (**b**), obtained by nanoindentation for 2.2 μm thick BiI_3_ films deposited on 211 Precision glass, 7059 Corning glass, Z-cut quartz, 〈111〉 silicon and fused silica.

It is a well-known fact in this field of study that the mechanical properties of substrates greatly affect hardness measurements. Thus, we carried out the nanoindentation measurements as a function of penetration depth, from 2% to approximately 50% of film thickness on BiI_3_ films (2.2 μm), so to evaluate this effect. Figure 4a demonstrates that the hardness of BiI_3_ films displays similar behavior in all substrates, indicating that the substrate does not greatly affect the measurements in a broad range of penetration depths, as expected for soft films deposited onto hard substrates^28^. Higher hardness values on the films’ surfaces were attributed to indentation size effect (which is to be expected in soft materials) and related to strain gradient plasticity^28,29^. An increase in the error bar for this region likely relates to defects in the surface. Since this region (~100 nm thick) is much thinner than the film thickness (2.2 μm), TIB measurements are not much affected by these defects. To use such films in devices, these defects can be reduced by the improvement in the deposition process, or by the use of passivation treatments, for instance. Hardness has a tendency to stabilize at the same value (0.76 ± 0.01 GPa) in penetration depths from 10% to 50% of film thickness, regardless of the substrate, indicating that this value is that which best represents the hardness of BiI_3_ thin films. P. M. Johns^30^ reported a value of 0.68 ± 0.014 GPa, obtained through dynamic indentation measurements, using atomic force microscopy in the (001) orientation of BiI_3_ crystal – which is comparable to the value we obtained for our microcrystalline films. 0.76 GPa may not be a high hardness value, but it is sufficient to permit the handling of a device without affecting its integrity; in fact, smaller values (inside the 0.2–0.45 GPa range) have been reported for few perovskite films^18^. The values for typical amorphous semiconductors (silicon and germanium) are, however, one order of magnitude higher^31,32^.

After a maximum penetration depth of 10% film thickness, one may observe a deviation of the reduced Young’s modulus (E_r_) (Fig. 4b). It is likely that this behavior is associated with the substrates’ mechanical properties^28^. The substrates’ hardness and Young’s modulus, determined using the same nanoindentation apparatus, are much higher than those of the films (see Methods section). These characteristics are positive for hardness determination, but the elastic constant of the thin films are more dramatically affected by the substrate than is the measurement of hardness, as reported by R. Saha *et al*.^28^. Considering this, one assumes that the average values obtained with indentation to the order of 10% penetration depth (27.1 ± 0.5 GPa) best represent the reduced Young’s modulus for BiI_3_ films. Sun *et al*.^18^ reported Young’s modulus for a number of lead halide perovskite single crystals in the 8–16 GPa range.

Table 1 summarizes the five thermomechanical properties of the BiI_3_ films that we obtained in this study. For the sake of comparison, the obtained biaxial [E/(1 − ν) = 19.6 ± 0.8 GPa] and reduced [E/(1 − ν^2^) = 27.1 ± 0.5 GPa] Young’s moduli are smaller than those theoretical (51.1 GPa and 40.9 GPa respectively) calculated by adopting Young’s modulus and Poisson’s ratio (38.3 GPa and 0.25 GPa respectively) obtained by DFT calculation for the R-3 monocrystalline phase of BiI_3_^33^. P. M. Johns reported experimental data for the bulk modulus (16.6 ± 5.5 GPa) determined by dynamic indentation using atomic force microscopy for the (001) surface of BiI_3_ crystal^30^. Since our films are microcrystalline, we expected the elastic moduli to be smaller than those in single crystals, due to a discontinuity in the structure at the microcrystalline grain boundaries, which also reduces the coordination number and thus, Young’s modulus. The small values of biaxial and reduced Young’s moduli permit a good amount of flexibility, as these indicate that a greater deformation in the material structure is possible when a force is applied, making BiI_3_ suitable for deposition on flexible substrates.

**Table 1:** Thermomechanical properties of BiI_3_ obtained by nanoindentation and thermally induced bending techniques: stress at 300 K (σ_300K_), hardness (H), coefficient of thermal expansion (CTE or α), and biaxial [E/(1 − ν)] and reduced [E/(1 − ν^2^)] Young’s moduli.

Stress *σ*_300*K*_ (MPa)	Hardness H (GPa)	Coefficient of thermal expansion α (10^−6^/°C)	Biaxial Young’s modulus E/(1 − ν) (GPa)	Reduced Young’s modulus E/(1 − ν^2^) (GPa)
28 ± 3	0.76 ± 0.01	31 ± 1	19.6 ± 0.8	27.1 ± 0.5

By using the biaxial [E_f_/(1 − ν_f_)] and reduced [E_f_/(1 − ν_f_^2^)] Young’s moduli of the films it would be possible, in principle, to separately calculate E_f_ and ν_f_^34^; however, it may only be possible to adopt this method for isotropic material. As observed here, Fig. 1a, BiI_3_ is an anisotropic material, with a preferential orientation in the (003) direction. The biaxial Young’s modulus extracted from TIB measurements relates to a direction parallel to the surface; and the reduced Young’s modulus obtained by nanoindentation is not limited to one specific orientation. Thus, applying a combination of these two methods for extracting Poisson’s ratio and Young’s modulus separately may provide incorrect data.

## Conclusions

The thermomechanical properties of BiI_3_ were determined for films prepared by thermal evaporation. The stress of the films was slightly tensile. By adopting different substrates with different thermal expansion coefficients, we calculated the coefficient of thermal expansion for the BiI_3_ films: (31 ± 1) × (10^−6^ °C^−1^), which is much greater than that of currently adopted substrates for solar cell manufacturers. The films presented a hardness of ~0.8 GPa, thus rendering them appropriate for electronic devices lacking protective coatings. Two elastic moduli related to Young’s modulus and Poisson’s ratio – the biaxial and reduced Young’s moduli – were obtained (19.6 ± 0.8 GPa and 27.1 ± 0.5 GPa respectively). By applying these two techniques therefore, we were able to obtain five useful thermomechanical properties in BiI_3_ films: stress, hardness, coefficient of thermal expansion, and reduced and biaxial Young’s moduli.

## Methods

### Sample preparation

Bismuth triiodide (BiI_3_) thin films were grown by the thermal evaporation of BiI_3_ powder (Sigma Aldrich, 99.999% trace metal basis) using a tungsten crucible, on double-sided polished substrates. The chamber base pressure was 2 × 10^5^ Torr, and 25 mm × 5 mm substrates were located above the crucible, at a distance of 9 cm. After deposition, film annealing occurred at 140 °C for 1 h, the final thickness being of about 2.2 μm. We used substrates with known mechanical properties: fused silica, 〈111〉 silicon, 211 Precision Glass, 7059 Corning Glass and Z-cut quartz, the properties of which are presented in Table 2.

**Table 2:** Properties of fused silica, 〈111〉 silicon, 7059 Corning glass, 211 Precision glass, and Z-cut quartz substrates: coefficient of thermal expansion (CTE), Young’s modulus (E_s_), Poisson’s ratio (ν_s_), E_s_/(1 − ν_s_^2^) calculated by using E_s_ and ν_s_, reduced Young’s modulus E_r_ = E_s_/(1 − ν_s_²) and hardness (H) experimentally determined by nanoindentation, and thickness.

Substrate	CTE (10^−6^/°C)	E_s_ (GPa)	ν_s_	E_s_/(1 − ν_s_²) (GPa)	E_r_ (GPa)	H (GPa)	Thickness (μm)
Fused silica	0.55^a^	71.7^a^	0.17^a^	74	72 ± 1	10.0 ± 0.4	500
〈111〉 Si	2.6^b^	168.9^b^	0.262^b^	181	165 ± 6	10.1 ± 0.3	380
7059	4.6^c^	68.9^c^	0.20^c^	72	71 ± 0.2	7.4 ± 0.1	390
211	7.4^d^	74.5^d^	0.22 ^d^	78	78 ± 2	7.4 ± 0.6	520
Z-cut Quartz	13.37^e^	76.5^e^	0.08^e^	77	109 ± 2	13.7 ± 0.5	500

^a^See ref.^39^; ^b^See ref.^40,41^; ^c^See ref.^42^; ^d^See ref.^43^; ^e^See ref.^44^.

### Optical and structural properties

We measured X-ray diffraction (XRD) with a Bruker D8 Advanced diffractometer, using a Cu Kα radiation source. Transmittance data were collected with a UV-Vis M51 spectrophotometer from Bel Photonics. The optical constants were recovered from the transmittance data using the PUMA (Pointwise Unconstrained Minimization Approach) package^35^. A FIB-SEM Nova 200 Nanolab spectrometer generated the michograph images. We collected the photoluminescence (PL) spectra by exciting the material with a 442 nm laser with an integration time of 60 s. The film thickness and curvature we obtained at room temperature using a Dektak 150 Surface Profiler.

### Stress, coefficient of thermal expansion and biaxial Young’s Modulus

**Figure 5:**
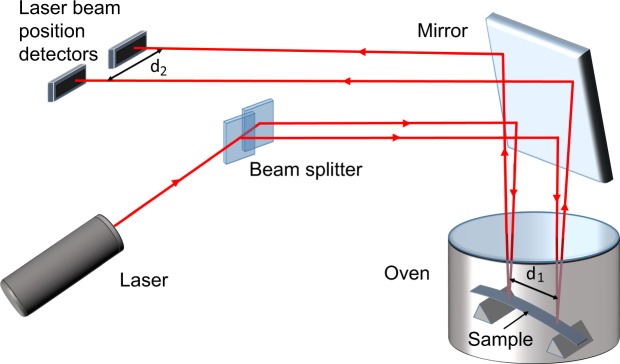
Scheme for the apparatus used in the thermally induced bending technique, to determine the curvature dependence of the film-substrate composite as a function of temperature.

The thermally induced bending technique (TIB) was utilized to determine film stress as a function of temperature (Fig. 5). Fundamentally speaking, a laser beam passes through a beam splitter, creating two beams that reach the film, these being reflected onto two position detectors, from which one can calculate the curvature radius (R) of the film-substrate composite. When the sample undergoes heating its curvature changes due to a difference between the CTE of the substrate and the film. Thus one may calculate the temperature dependence of stress [σ(T)] in the film, using the modified Stoney equation^36,37^:

1 $${\rm{\sigma }}({\rm{T}})=[\frac{{{\rm{E}}}_{{\rm{s}}}}{1-{{\rm{\nu }}}_{{\rm{s}}}}]\frac{{{\rm{t}}}_{{\rm{s}}}^{2}}{6{{\rm{t}}}_{{\rm{f}}}}(\frac{1}{{\rm{R}}({\rm{T}})}-\frac{1}{{{\rm{R}}}_{0}}),$$

where *t* is the thickness, and subscripts *s* and *f* respectively refer to the substrate and film. *1/R* and *1/R*_0_ refer to the curvature of the film-substrate sample, and the substrate before deposition, respectively.

The derivative of stress with respect to the temperature dσ/dT relates to the substrate (α_s_) and the film (α_f_) coefficients of thermal expansion by Eq. (2):

2 $$\frac{{\rm{d}}{\rm{\sigma }}}{{\rm{dT}}}=[\frac{{{\rm{E}}}_{{\rm{f}}}}{1-{{\rm{\nu }}}_{{\rm{f}}}}]({{\rm{\alpha }}}_{{\rm{s}}}-{{\rm{\alpha }}}_{{\rm{f}}}).$$

Using two substrates with different thermal expansion coefficients one can solve Eq. (2) in order to extract the CTE and Young’s biaxial modulus [E_f_/(1 − ν_f_)] of a given film^12^. Thick films (having a thickness of at least 1 micron) are required for these measurements due to a limitation in the technique, which demands that one bends a thick substrate (thickness of about 500 μm) inside a small temperature range (of less than 100 °C). In light of this, the thicker the film the more appropriate it is for this measurement. In addition, measurements are made by the use of two laser beams 10 mm apart, thus requiring a homogenous film distributed along the substrate.

For these measurements, we adopted a 30 mW helium-neon laser and heated the samples in an argon atmosphere from room temperature to 130 °C, at 6.3 °C/min, the samples remaining at this temperature for 20 minutes. Following this, they underwent cooling to room temperature, followed by heating and then further cooling. The stress temperature dependence was obtained during the two cooling cycles, from room temperature to 80 °C. Five BiI_3_ films were deposited on each type of substrate in different deposition runs, to reduce uncertainty regarding the thermomechanical properties encountered in the extracts.

### Hardness and reduced Young’s Modulus

We carried out nanoindentation measurements of the BiI_3_ films using a Berkovich diamond tip (NanoTest-300) with a dwell time of 20 seconds, and the load-displacement curves we analyzed through the Oliver and Pharr method^13^. Detailed description of the system and procedures appear elsewhere^38^. Hardness (H) and reduced Young’s modulus (E_s_/(1 − ν_s_^2^) of all substrates were also determined by nanoindentation (Table 2). The values for glassy (amorphous) substrates (fused silica, 211 Precision Glass and 7059 Corning Glass) are very close to the calculated ones using reported data for Young’s modulus (E_s_) and Poisson’s ratio (ν_s_). This is unsurprising, since these substrates are isotropic. Conversely, for crystalline substrates (〈111〉 silicon and Z-cut quartz), which are anisotropic, the experimental reduced Young’s modulus determined by nanoindentation is different from the one calculated. This is because indentation with a Berkovich diamond tip involves several crystalline orientations with various mechanical properties.
